# Association Between Serum Ferritin Levels and the Risk of Kidney Stones in Patients With Type 2 Diabetes Mellitus: A Cross-Sectional Study

**DOI:** 10.1155/ije/2454470

**Published:** 2025-11-26

**Authors:** Wenya Mo, Lei Chen, Jingran Bian, Qifei Dong, Ling Wang, Lulu Zhu, Mei Zhao

**Affiliations:** ^1^Department of Urology, The First Affiliated Hospital of USTC, Division of Life Sciences and Medicine, University of Science and Technology of China, Hefei, Anhui, China; ^2^School of Nursing, Anhui Medical University, Hefei, Anhui, China; ^3^Affiliated Anhui Provincial Hospital of Anhui Medical University, Hefei, Anhui, China

**Keywords:** kidney stones, risk factors, serum ferritin, Type 2 diabetes mellitus

## Abstract

**Objectives:**

Prior research has identified a significant correlation between elevated serum ferritin (SF) levels and comorbidities in Type 2 diabetes mellitus (T2DM) patients. However, the association between SF levels and kidney stone occurrence in T2DM remains unexplored. This study aimed to assess the relationship between SF levels and kidney stone risk in T2DM patients.

**Methods:**

This study collected data from 50,583 patients with T2DM who received treatment at the First Affiliated Hospital of University of Science and Technology of China from December 2015 to December 2023. Ultimately, 1024 eligible patients were included for analysis. Multivariable logistic regression models were used to determine the odds ratio (OR) and 95% confidence interval (95% CI) for the association between SF and kidney stones. A multivariable-adjusted restricted cubic spline model was constructed to establish the OR curves to examine the possible nonlinear dose–response association between SF and kidney stones.

**Results:**

Of 1024 patients included in this study (mean age, 56.31 ± 9.82 years-old; 686 [67.0%] male), 148 (14.5%) reported having kidney stones, while 876 (85.5%) did not. After adjusting for potential confounders, the SF levels were associated with kidney stones (OR = 1.001; 95% CI, 1.000–1.002; *p* < 0.001). Participants in the highest quartile (Q4) of SF levels (333.60 ≤ SF ≤ 1867.00 ng/mL) had an adjusted OR for kidney stones of 2.901 (95% CI, 1.710–4.901; *p* trend < 0.001) compared to those in the lowest quartile (Q1) (6.20 ≤ SF ≤ 99.35 ng/mL). The multivariable restricted cubic spline showed a nonlinear association between SF levels and kidney stones (*p*=0.033). Subgroup analyses showed that SF levels were associated with kidney stones in male (OR = 2.04; 95% CI, 1.06–4.14), individuals ≤ 60 years-old of age (OR = 2.34; 95% CI, 1.21–4.73), with no smoke history (OR = 2.00; 95% CI, 1.06–3.85).

**Conclusion:**

Elevated SF levels are associated with kidney stones in T2DM patients.

## 1. Introduction

Type 2 diabetes mellitus (T2DM) is a chronic metabolic disorder characterized by hyperglycemia, resulting in various complications over time, representing a significant public health concern affecting both physical and mental well-being [1, 2]. Kidney stones, influenced by environmental, dietary, and genetic factors, have an incidence rate ranging from 6% to 12%, with a recurrence rate of up to 50% within 5 years, significantly compromising human health [3, 4]. Previous research suggests that individuals with T2DM have an increased risk of both primary and recurrent kidney stone formation compared to those without diabetes, with diabetes identified as a risk factor for nephrolithiasis [5, 6]. Additionally, some research has shown that individuals with T2DM are more susceptible to bacterial infections when they have concurrent kidney stones. Infections may result in poorly controlled hyperglycemia, exacerbating the infection, thus establishing a vicious cycle [7]. The management of nephrolithiasis in individuals with T2DM has received considerable attention due to the elevated healthcare expenses and societal impact. Nevertheless, the risk factors for nephrolithiasis in this population remain incompletely understood.

Serum ferritin (SF) serves as a marker reflecting the body's iron stores and is a sensitive indicator for assessing iron deficiency or overload. Additionally, ferritin functions as an inflammatory marker, with its concentration elevated in response to inflammation and various diseases [8]. Elevated SF levels can increase oxidative stress, exacerbating lipid peroxidation and activating intracellular stress-sensitive signaling pathways, leading to the production of inflammatory factors [9]. Prior research has shown a close association between SF and the onset and progression of chronic low-grade inflammatory conditions including insulin resistance, obesity, metabolic syndrome, and diabetes [10–12]. Individuals with T2DM demonstrate markedly elevated SF levels compared to those without diabetes. Moreover, research has identified a significant correlation between elevated SF levels and the presence of concurrent comorbidities in T2DM patients [13]. However, research investigating the relationship between SF and the risk of nephrolithiasis in individuals with T2DM is currently limited. Therefore, this study aims to investigate whether elevated SF levels pose a risk factor for nephrolithiasis in T2DM patients, contributing to the scientific basis for disease prevention and treatment.

## 2. Patients and Methods

### 2.1. Study Design and Population

**Figure 1:**
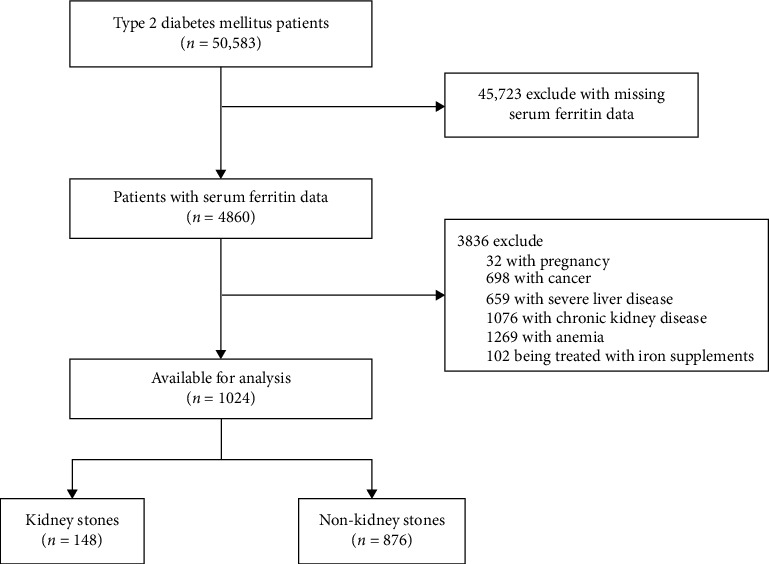
Flow diagram of participants in study.

The cross-sectional study collected data from 50,583 patients with T2DM who received treatment at the First Affiliated Hospital of University of Science and Technology of China (Anhui Provincial Hospital) from December 2015 to December 2023. Informed consent requirements were waived since this was a retrospective analysis of data extracted from participants' medical records. The exclusion criteria were as follows: (1) Lack of SF testing or missing urinary system imaging data during hospitalization; (2) ongoing iron supplement treatment; (3) presence of conditions like cancer, severe liver disease, chronic kidney disease (CKD) defined as eGFR ≤ 90 mL/min/1.73 m^2^, or other coexisting factors such as pregnancy or rheumatic connective tissue disease; (4) anemia patients (hemoglobin < 120 g/L in males, < 110 g/L in females); (5) with congenital anomalies in the kidney or urinary tract, renal tumors, nephrectomy, dialysis, or renal transplantation. Patient data, including demographics, physical and laboratory measurement, and renal and urinary tract imaging results, were extracted from medical records. Kidney stone occurrence in urological examinations served as the outcome indicator. A total of 1024 patients were included according to the exclusion criteria (Figure 1), with 148 having kidney stones and 876 without. This cross-sectional study was reported in strict accordance with the STROBE Checklist, which is provided as supporting information (Supporting Information S1).

### 2.2. T2DM and Kidney Stones

According to the Chinese Guideline for the Prevention and Treatment of Type 2 Diabetes Mellitus [14]: diabetes diagnosis requires the presence of symptoms (polydipsia, polyuria, polyphagia, unexplained weight loss) along with one of the following: (1) Random blood glucose ≥ 11.1 mmol/L (random blood glucose refers to blood glucose at any time of the day without considering the last meal and cannot be used to diagnose impaired fasting glucose or impaired glucose tolerance); (2) fasting plasma glucose (FPG) ≥ 7.0 mmol/L (note: fasting state refers to at least 8 h without eating); (3) glycated hemoglobin (HbA1c) ≥ 6.5%; and (4) blood glucose at 2 h after glucose load ≥ 11.1 mmol/L. For asymptomatic individuals, repeat testing on another day is necessary for confirmation of diagnosis.

The study's main outcome was the incidence of kidney stones, identified based on urinary tract ultrasonography and helical computed tomography results performed by well-trained radiologists using the same model of machine in the hospital [15]. Stone characteristics (e.g., length, number, and location) were recorded, with the size of the stone being considered as the length of the largest stones in cases of multiple stones.

### 2.3. Covariates

Based on previous study [16], the covariates included age, sex, body mass index (BMI), history of smoking and drinking, systolic blood pressure (SBP), diastolic blood pressure (DBP), duration of diabetes, FPG, glycosylated hemoglobin (HbA1c), SF, hemoglobin (Hb), white blood cells (WBCs), albumin (Alb), platelets (PLTs), alanine aminotransferase (ALT), aspartate aminotransferase (AST), alkaline phosphatase (ALP), total bilirubin (TBil), direct bilirubin (DBil), glomerular filtration rate (eGFR), uric acid (UA), total cholesterol (TC), triglycerides (TGs), low-density lipoprotein cholesterol (LDL-c), high-density lipoprotein cholesterol (HDL-c). History of smoking and drinking alcohol was recorded as never or smoker or drinker. Estimated glomerular filtration rate (eGFR) was calculated using the following formula: 194 × Cr^−1.094^ × Age^−0.287^ (for female patients, multiplied by 0.739). BMI was calculated as weight (kg)/height (m)^2^. SF levels and other laboratory measurements of all patients were uniformly tested in hospital laboratories.

### 2.4. Statistical Analyses

During the data cleaning phase, the missing data of each variable were first assessed via frequency analysis. The results revealed that variables including age (missing rate: 3.4%), BMI (missing rate: 6.5%), and diabetes duration (missing rate: 8.2%) had missing values, with the missing rate of all variables being less than 20%. Correlation analysis indicated that the missing data mechanism conformed to missing at random (MAR); therefore, multiple imputation was employed to handle the missing data in this study. The normality of continuous variables was assessed using the Shapiro–Wilk tests. Normally distributed continuous variables were described as mean ± standard deviation (SD). Non-normal distribution continuous variables were described as medians [interquartile ranges (IQR)] and compared using Mann–Whitney or Kruskal–Wallis tests. Categorical variables were presented as frequencies (percentages) and compared using chi-square tests. Multivariable logistic regression models were used to determine the odds ratio (OR) and 95% confidence interval (95% CI) for the association between SF and kidney stones. First, univariate logistic regression analysis was performed for each independent variable and the outcome variable, and variables were screened based on statistical significance (*p* < 0.05). Subsequently, the screened variables were included in the multivariable logistic regression model. Model 1 was adjusted for age, sex, and BMI. Model 2 was adjusted for the potential confounding variables included in Model 1 and history of drinking, DBP, SF, Hb, PLT, DBil, eGFR, Alb and HDL-c. In the logistic regression models, SF scores were categorized into quartiles, and a trend test was conducted to enhance result robustness. Additionally, a multivariable-adjusted restricted cubic spline model was developed to establish OR curves with 3 knots and the reference value was defined as the median of the lowest quartile (Q1) of SF levels (6.20 ≤ SF ≤ 99.35 ng/mL), aiming to explore the potential nonlinear dose–response relationship between SF and kidney stones. Finally, to determine whether the relationship between SF and kidney stones is stable across populations, interaction and subgroup analyses were performed according to sex, age (≤ 60 versus > 60 years), and BMI (≤ 25 versus > 25 kg/m^2^). Statistical analysis was conducted using IBM SPSS Statistics 22.0 (IBM Co., Armonk, New York, USA) and R software, version 4.2.3 (https://cloud.r-project.org/). Two-tailed *p* values less than 0.05 were considered statistically significant.

## 3. Results

### 3.1. Baseline Characteristics of Study Subjects

The data of 1024 patients with T2DM were analyzed. In all, 148 participants (14.5%) had kidney stones, while 876 (85.5%) did not. The total SF level ranged from 6.2 to 1867 ng/mL.  Table 1 presents the baseline characteristics of the 1024 study participants according to their SF score quartile. The mean age of the study subjects was 56.31 ± 9.82 years old, and 338 (33%) participants were women. Compared with the Q1 (≤ 6.2 to ≤ 99.35), the occurrence of kidney stones was associated with higher SF levels in the Q4 (< 333.6 to ≤ 1867) (64 [25%] versus 24 [9.4%], respectively). The SF levels were higher in men than in women females (226 [88.3%] versus 30 [11.7%], respectively), in nonsmokers were compared with former smokers and current smokers (165 [64.5%] versus 91 [35.5%], respectively), and in nondrinkers were compared with former drinkers and current drinkers (176 [68.8%] versus 80 [31.2%], respectively).

### 3.2. Association Between SF and Kidney Stones in Patients With T2DM

**Figure 2:**
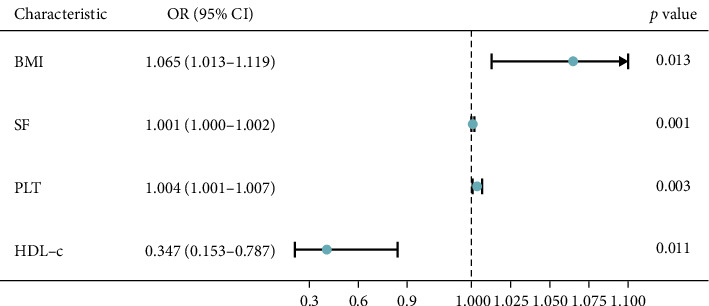
Risk factors for kidney stones in T2DM by logistic regression analysis. Abbreviations: body mass index (BMI), serum ferritin (SF), platelets (PLT), high-density lipoprotein cholesterol (HDL-c).

A multivariable logistic regression model was used to analyze the risk factors for kidney stones. After adjusting for all factors with a *p*-value less than 0.05 in the univariate logistic regression includes sex, age, BMI, history of alcohol, DBP, SF, Hb, PLT, DBil, eGFR, Alb, and HDL-c; BMI, SF, PLT, and HDL-c were identified as significant independent risk factors for kidney stones; the ORs (95% CI) were 1.065 (1.013–1.119), 1.001 (1.000–1.002), 1.004 (1.001–1.007), and 0.347 (0.153–0.787), respectively (all *p* < 0.05) (Figure 2).

In the logistic regression model, for multicategory variables, the reference category for gender was set as male, with an OR of 1.501 and a *p* value of 0.074; the reference category for smoking status was nonsmoking, with an OR of 0.927 and a *p* value of 0.726; the reference category for drinking status was nondrinking, with an OR of 0.730 and a *p* value of 0.154. Multivariable regression analysis confirmed that SF was a relatively independent risk factor for kidney stones in T2DM patients. This association was maintained when the SF scores were transformed into a categorical variable as quartiles, with the tangent point values of 99.35, 185.74, and 333.60, respectively. In the unadjusted model, Q4 of SF levels (333.60 ≤ SF ≤ 1867.00 ng/mL) was found to have a significantly higher risk of developing kidney stones than Q1 of SF levels (6.20 ≤ SF ≤ 99.35 ng/mL) (OR = 3.222; 95% CI 1.942–5.348; *p* trend < 0.001), minimally adjusted Model 1 (OR = 3.120; 95% CI 1.876–5.190; *p* trend = 0.007) and fully adjusted Model 2 (OR = 2.901; 95% CI 1.710–4.901; *p* trend < 0.001), while no significant difference was observed between Q1, Q2, and Q3 (Table 2).

### 3.3. Nonlinearity Analysis and Subgroup Analyses

**Figure 3:**
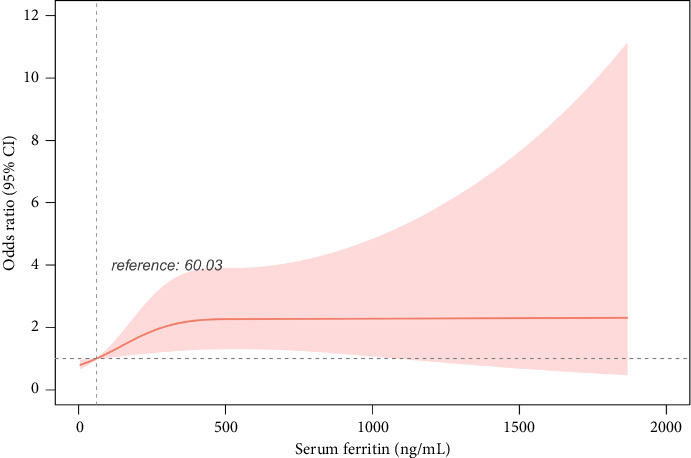
The nonlinear relationship between SF and kidney stones in T2DM. The restricted cubic spline model was adjusted for sex, age, BMI, history of alcohol, DBP, SF, Hb, PLT, DBil, eGFR, Alb, and HDL-c. Abbreviations: body mass index (BMI), diastolic blood pressure (DBP), serum ferritin (SF), hemoglobin (Hb), platelets (PLT), direct bilirubin (DBil), estimated glomerular filtration rate (eGFR), albumin (Alb), high-density lipoprotein cholesterol (HDL-c).

Additionally, the association between SF and kidney stones showed nonlinearity (*p*=0.033) in the restricted cubic spline model (Figure 3). The study findings showed that the risk of developing kidney stones increased significantly with elevated SF levels; however, this risk tended to stabilize once SF exceeded 333.2 ng/mL. Using this inflection point as the cutoff value, we conducted a binary logistic regression analysis. The results revealed that for participants with SF ≥ 333.2 ng/mL, the adjusted OR for kidney stone development was 2.656 (95% CI: 1.325–5.326; *p* < 0.001). In contrast, for participants with SF < 333.2 ng/mL, the adjusted OR for kidney stone development was 1.003 (95% CI: 1.001–1.005; *p*=0.003).

**Figure 4:**
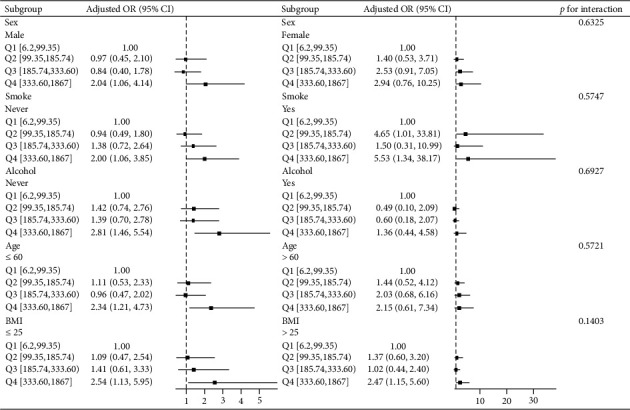
Association between SF and kidney stones according to the general characteristics. Except for the stratification factor itself, the stratifications were adjusted for all variables (sex, age, BMI, history of alcohol, history of smoke, DBP, SF, Hb, PLT, DBil, eGFR, Alb, and HDL-c). Abbreviations: body mass index (BMI), diastolic blood pressure (DBP), serum ferritin (SF), hemoglobin (Hb), platelets (PLT), direct bilirubin (DBil), estimated glomerular filtration rate (eGFR), albumin (Alb), high-density lipoprotein cholesterol (HDL-c).

Subgroup analyses (Figure 4) revealed SF associations with kidney stones in male (OR, 2.04; 95% CI, 1.06–4.14), individuals ≤ 60 years old (OR, 2.34; 95% CI, 1.21–4.73), nonsmokers (OR, 2.00; 95% CI, 1.06–3.85), nondrinkers (OR, 2.81; 95% CI, 1.46–5.54), smokers (OR, 5.53; 95% CI, 1.34–38.17), and with a BMI ≤ 25 kg/m^2^ (OR, 2.54; 95% CI, 1.13–5.95), BMI > 25 kg/m^2^ (OR, 2.47; 95% CI, 1.15–5.60). There was no association between SF and kidney stones in female or patients over 60 years old.

## 4. Discussion

This study enrolled 50,583 patients with T2DM, from whom 1024 eligible individuals were selected for analysis to investigate the risk factors associated with kidney stone development. Multivariable logistic regression analysis identified elevated BMI, PLT, SF, and decreased HDL-c as relatively independent risk factors for concomitant kidney stones in T2DM patients. Previous studies have established an association between high BMI and an elevated risk of kidney stones in individuals with diabetes [17]. Dagfinn et al. conducted a systematic review and meta-analysis, which revealed a positive correlation between obesity and the risk of kidney stones in diabetic patients [5]. Mao et al. conducted a cross-sectional study, collecting clinical data from 4008 adult diabetic participants in the National Health and Nutrition Examination Survey (NHANES) database spanning from 2007 to 2018. Employing both univariate and multivariate logistic regression analyses to explore the relationship between BMI and kidney stones, founding that high BMI was associated with an increased risk of kidney stones in diabetic participants [18], consistent with our study findings. Moreover, our results corroborated the association between high BMI and the formation of kidney stones in T2DM patients. This association may be attributed to physiological changes, such as increased uric acid secretion and urine concentration induced by obesity. Elevated PLT levels are associated with inflammatory responses and thrombus formation, both of which may contribute to the formation of urinary tract stones [19]. Our findings suggest that increased PLT levels may reflect elevated levels of inflammation in T2DM patients, thus potentially increasing the risk of kidney stone formation. Further mechanistic studies are warranted to elucidate the precise relationship between PLT levels and the formation of kidney stones. HDL-c is a crucial component of lipid metabolism and is associated with chronic inflammation and metabolic disorders. Our study identified a correlation between decreased HDL-c levels and an increased risk of kidney stones in T2DM patients, suggesting that abnormal lipid metabolism may be another significant factor contributing to stone formation. Therefore, enhancing the monitoring and management of lipid metabolism in T2DM patients holds promise for mitigating the risk of kidney stones.

Iron, one of the most abundant trace elements in the body, is predominantly obtained from dietary intake and the breakdown of senescent red blood cells, and it is distributed throughout all tissues of the human body. Ferritin, as an acute-phase protein, is closely linked to the onset and progression of diabetes [20]. Yeap et al. discovered that elevated SF levels were a relatively independent predictor for diabetes and could be used to forecast the risk of diabetes onset [21]. In a case–control study comprising 13,848 subjects, Kim et al. demonstrated that elevated SF concentration was an independent risk factor for insulin resistance, metabolic syndrome, impaired fasting glucose, and T2DM in male individuals [22]. Chen et al. also reported that elevated SF levels were linked to heightened insulin resistance and the risk of developing T2DM [12]. Furthermore, studies have unveiled a significant correlation between elevated SF levels and the occurrence of various diseases and complications in association with T2DM. Shang et al. findings suggest that hyperferritinemia is linked to elevated C-reactive protein levels, diabetic retinopathy, and coronary heart disease incidence in T2DM patients. Additionally, the study observed that elevated SF levels in males were correlated with dyslipidemia, hepatic dysfunction, and microalbuminuria [13]. This study identified an association between elevated SF levels and the occurrence of kidney stones in T2DM patients, possibly attributable to the shared inflammatory processes underlying both conditions [23–25]. Furthermore, studies have shown that T2DM commonly presents as a condition of chronic low-grade inflammation, and the development of T2DM is positively associated with low-grade inflammation [26]. Inflammation is widely acknowledged as a significant contributor to the development of insulin resistance, thereby substantially contributing to the progression of T2DM and playing a role in its anticipated complications [27]. Likewise, abundant evidence has confirmed the mediating role of inflammatory markers in the onset of Type 2 diabetes and its eventual long-term complications, connecting the initiation of diabetes to conditions arising from inflammatory mechanisms [28]. In recent years, a growing body of research has also affirmed the significant role of inflammatory damage responses in the occurrence and progression of kidney stones, suggesting a close association between inflammation, oxidative stress responses, and the onset of nephrolithiasis [23, 29–31]. The association between ferritin and inflammation may explain why elevated SF levels elevate the risk of kidney stone formation in patients with T2DM.

After adjusting for potential confounders, this study identified elevated SF levels as a relatively independent risk factor for kidney stones in individuals with T2DM. Additionally, the dose–response analysis unveiled a nonlinear association between SF levels and kidney stone occurrence. The risk of developing kidney stones significantly increased with higher SF levels, and this trend plateaued when SF exceeded 333.2 ng/mL (OR = 2.656; 95% CI, 1.325–5.326). The association remained robust in sensitivity analyses and subgroup analyses. Elevated serum iron levels may be associated with increased iron intake or abnormalities in iron metabolism among patients. Hence, monitoring and interventions aimed at managing serum iron levels in patients with T2DM may be beneficial in decreasing the incidence of kidney stones.

This study had several limitations. Firstly, it was conducted within a Chinese population, and further research is warranted to validate the applicability of our findings to other populations. Secondly, despite constructing a multivariable logistic regression model and conducting subgroup and sensitivity analyses to mitigate potential confounding effects on the association between SF levels and kidney stones, residual confounding remains a possibility. Lastly, due to the cross-sectional design of this study, causality between SF levels and kidney stones among patients with T2DM cannot be established. Thus, longitudinal studies are required to ascertain the causal relationship between them. Subsequent research can delve deeper into the association between SF and the occurrence of kidney stones in individuals with T2DM, elucidating its underlying physiological and pathological mechanisms. This endeavor holds promise for offering novel interventions and preventive strategies in clinical practice, with the goal of mitigating the risk of kidney stones in individuals with T2DM and alleviating the disease burden. Continued in-depth research may provide a better understanding of the pathogenesis of kidney stones in patients with T2DM, leading to more precise strategies for their prevention and treatment.

## 5. Conclusion

The present study identified elevated SF levels as a relatively independent risk factor for kidney stones in individuals with T2DM. Additionally, the dose–response analysis unveiled a nonlinear association between SF levels and kidney stone occurrence. Attention should be paid to, and intervention should be made to SF levels in such patients to reduce the risk of kidney stones. This may provide a scientific basis for the prevention and treatment of such diseases.

## Figures and Tables

**Table 1:** Characteristics of participants stratified by SF quartiles.

Characteristics^a^	Total (*N* = 1024) (≤ 6.2 to ≤ 1867)	Q1 (*N* = 256) (≤ 6.2 to ≤ 99.35)	Q2 (*N* = 256) (< 99.35 to ≤ 185.74)	Q3 (*N* = 256) (< 185.74 to ≤ 333.6)	Q4 (*N* = 256) (< 333.6 to ≤ 1867)	*p* value
Gender (%)						< 0.001
Male	686 (67.0)	117 (45.7)	151 (59.0)	192 (75.0)	226 (88.3)	
Female	338 (33.0)	139 (54.3)	105 (41.0)	64 (25.0)	30 (11.7)	
Age (years)	56.31 ± 9.82	58.68 ± 8.83	57.77 ± 9.75	56.03 ± 9.21	52.75 ± 10.4	< 0.001
BMI (kg/m^2^)	25.32 ± 3.52	25.18 ± 3.71	24.9 ± 3.15	25.35 ± 3.26	25.84 ± 3.52	0.022
History of smoking (%)						< 0.001
Nonsmoker	759 (74.1)	221 (86.3)	204 (79.7)	169 (66.0)	165 (64.5)	
Smoker	265 (25.9)	35 (13.7)	52 (20.3)	87 (34.0)	91 (35.5)	
History of alcohol (%)						< 0.001
Nonalcohol	805 (78.6)	219 (85.5)	218 (85.2)	192 (75.0)	176 (68.8)	
Drinker	219 (21.4)	37 (14.5)	38 (14.8)	64 (25.0)	80 (31.2)	
Kidney stones (%)						< 0.001
No	876 (85.5)	232 (90.6)	227 (88.7)	225 (87.9)	192 (75)	
Yes	148 (14.5)	24 (9.4)	29 (11.3)	31 (12.1)	64 (25)	
SBP (mmHg)	133.02 ± 16.72	133.54 ± 17.1	137.02 ± 17.08	133.61 ± 16.13	132.88 ± 16.71	0.106
DBP (mmHg)	83.89 ± 10.66	87.79 ± 9.78	82.26 ± 10.07	85.0 ± 10.5	85.47 ± 10.86	0.001
Duration of diabetes (y)	10.02 ± 4.81	10.87 ± 4.63	10.05 ± 4.64	9.46 ± 4.65	9.68 ± 4.12	0.005
FPG (mmol/L)	8.49 ± 2.72	7.55 ± 2.37	8.55 ± 2.63	8.8 ± 3.15	9.04 ± 2.44	< 0.001
HbA1c (%)	8.09 ± 1.95	7.64 ± 1.67	8.19 ± 1.91	8.12 ± 1.9	8.5 ± 2.17	< 0.001
Hb (g/L)	137.04 ± 13.47	131.7 ± 11.23	134.62 ± 12.85	139.55 ± 13.16	142.29 ± 13.94	< 0.001
WBC (10^9^/L)	6.07 ± 1.05	5.93 ± 1.5	6.0 ± 1.51	6.12 ± 1.58	6.22 ± 1.57	0.157
Alb (g/L)	40.17 ± 3.94	39.87 ± 3.64	39.81 ± 3.32	40.4 ± 4.42	40.56 ± 4.42	0.067
PLT (10^9^/L)	199.75 ± 63.82	206.45 ± 63.31	198.45 ± 62.35	197.65 ± 63.6	196.46 ± 65.83	0.003
ALT (IU/L)	20.25 (14.50, 30.98)	17.55 (13.42, 25)	19.4 (14.2, 27.8)	22 (14.1, 33.97)	25 (17, 44)	< 0.001
AST (IU/L)	20.00 (16.00, 25.00)	19.05 (16, 23.4)	19 (15.67, 23.97)	19.95 (15.8, 24)	23 (17, 32.6)	0.002
ALP (μmol/L)	75.00 (62.00, 89.00)	74 (60.25, 87.75)	75 (62, 89.75)	75.5 (61.25, 89)	74 (63, 91.75)	0.591
TBil (μmol/L)	12.80 (9.63, 16.40)	11.76 (8.7, 14.8)	12.8 (10.4, 16.1)	13.3 (9.72, 17.4)	13.95 (9.9, 17.6)	0.003
DBil (μmol/L)	3.50 (2.40, 4.50)	2.8 (2.1, 3.9)	3.5 (2.5, 4.2)	3.6 (2.5, 4.97)	4.2 (2.9, 5.1)	< 0.001
eGFR (mL/min/1.73 m^2^)	124.96 ± 30.60	119.86 ± 25.64	119.83 ± 28.83	125.48 ± 27.89	134.67 ± 34.57	< 0.001
UA (mmol/L)	301 (248, 370.75)	288 (236.7355.4)	291 (237, 355.02)	308.2 (250, 379)	331 (264, 391)	< 0.001
TC (mmol/L)	4.42 ± 1.11	4.28 ± 1.03	4.34 ± 0.9	4.46 ± 1.05	4.61 ± 1.36	< 0.001
TG (mmol/L)	1.8 0 (1.23, 1.87)	1.62 (1.1, 1.79)	1.56 (1.07, 1.83)	1.79 (1.26, 1.84)	1.79 (1.65, 2.31)	0.001
LDL-c (mmol/L)	2.47 ± 0.74	2.49 ± 0.76	2.43 ± 0.70	2.52 ± 0.71	2.54 ± 0.76	0.098
HDL-c (mmol/L)	0.99 ± 0.27	1.05 ± 0.28	1.01 ± 0.28	0.97 ± 0.22	0.98 ± 0.27	< 0.001

*Note:* HbA1c = glycosylated hemoglobin, Hb = hemoglobin, Alb = albumin, PLT = platelets, ALT = alanine aminotransferase, AST = aspartate aminotransferase, ALP = alkaline phosphatase, TBil = total bilirubin, DBil = direct bilirubin, TG = triglycerides.

Abbreviations: BMI = body mass index, DBP = diastolic blood pressure, eGFR = estimated glomerular filtration rate, FPG = fasting plasm glucose, HDL-c = high-density lipoprotein cholesterol, LDL-c = low-density lipoprotein cholesterol, SBP = systolic blood pressure, SF = serum ferritin, TC = total cholesterol, UA = uric acid, WBC = white blood cells.

^a^Data are *n* (%), mean ± SD, or median (interquartile range).

**Table 2:** Association between quartiles of SF and prevalent kidney stones in T2DM.

Characteristics variable	Univariate analysis OR (95% CI)	Model 1^∗^ OR (95% CI)	Model 2^∗∗^ OR (95% CI)
Q1	Reference	Reference	Reference
Q2	1.235 (0.698–2.186)	1.264 (0.713–2.243)	1.239 (0.695–2.212)
Q3	1.332 (0.758–2.340)	1.327 (0.754–2.336)	1.291 (0.729–2.286)
Q4	**3.222 (1.942–5.348)**	**3.120 (1.876–5.190)**	**2.901 (1.71–4.901)**
*p*_for⁣trend_	< 0.001	0.007	< 0.001

*Note:* Hb = hemoglobin, PLT = platelets, DBil = direct bilirubin, Alb = albumin. The bold values in the table indicate a statistically significant difference between the Q1 and Q4 groups after quartile categorization of serum ferritin levels, following adjustment for different models (*p* < 0.05). This confirms the statistical significance of the observed results.

Abbreviations: BMI = body mass index, DBP = diastolic blood pressure, eGFR = estimated glomerular filtration rate, HDL-c = high-density lipoprotein cholesterol, SF = serum ferritin.

^∗^Model 1: adjusted for age, sex, BMI.

^∗∗^Model 2: adjusted for age, sex, BMI, history of alcohol, DBP, SF, Hb, PLT, DBil, eGFR, Alb, and HDL-c.

## Data Availability

The dataset analyses during the current study are available from the corresponding author.

## Supporting Information

Supporting Information S1: The STROBE checklist for cross-sectional studies, which outlines the reporting standards followed in the preparation of this manuscript.

## References

[1] Tinajero M. G., Malik V. S. (2021). An Update on the Epidemiology of Type 2 Diabetes: a Global Perspective. *Endocrinology and Metabolism Clinics of North America*.

[2] Li Y., Teng D., Shi X. (2020). Prevalence of Diabetes Recorded in Mainland China Using 2018 Diagnostic Criteria from the American Diabetes Association: National Cross Sectional Study. *BMJ*.

[3] Zeng G., Mai Z., Xia S. (2017). Prevalence of Kidney Stones in China: an Ultrasonography Based cross-sectional Study. *BJU International*.

[4] Peerapen P., Thongboonkerd V. (2023). Kidney Stone Prevention. *Advances in Nutrition*.

[5] Aune D., Mahamat-Saleh Y., Norat T., Riboli E. (2018). Body Fatness, Diabetes, Physical Activity and Risk of Kidney Stones: a Systematic Review and meta-analysis of Cohort Studies. *European Journal of Epidemiology*.

[6] Weinberg A. E., Patel C. J., Chertow G. M., Leppert J. T. (2014). Diabetic Severity and Risk of Kidney Stone Disease. *European Urology*.

[7] Wei W., Leng J., Shao H., Wang W. (2015). Diabetes, a Risk Factor for Both Infectious and Major Complications After Percutaneous Nephrolithotomy. *International Journal of Clinical and Experimental Medicine*.

[8] Kell D. B., Pretorius E. (2014). Serum Ferritin is an Important Inflammatory Disease Marker, as it is Mainly a Leakage Product from Damaged Cells. *Metallomics*.

[9] Namaste S. M., Rohner F., Huang J. (2017). Adjusting Ferritin Concentrations for Inflammation: Biomarkers Reflecting Inflammation and Nutritional Determinants of Anemia (BRINDA) Project. *The American Journal of Clinical Nutrition*.

[10] Pompano L. M., Correa-Burrows P., Burrows R., Blanco E., Lozoff B., Gahagan S. (2022). Adjusting Ferritin Concentrations for Nonclinical Inflammation in Adolescents with Overweight or Obesity. *The Journal of Pediatrics*.

[11] Yu L., Yan J., Zhang Q. (2020). Association Between Serum Ferritin and Blood Lipids: Influence of Diabetes and hs-CRP Levels. *Journal of Diabetes Research*.

[12] Chen L., Li Y., Zhang F., Zhang S., Zhou X., Ji L. (2018). Elevated Serum Ferritin Concentration is Associated with Incident Type 2 Diabetes Mellitus in a Chinese Population: a Prospective Cohort Study. *Diabetes Research and Clinical Practice*.

[13] Shang X., Zhang R., Wang X., Yao J., Zhao X., Li H. (2022). The Relationship of Hyperferritinemia to Metabolism and Chronic Complications in Type 2 Diabetes. *Diabetes, Metabolic Syndrome and Obesity: Targets and Therapy*.

[14] Jia W., Weng J., Zhu D. (2019). Standards of Medical Care for Type 2 Diabetes in China 2019. *Diabetes*.

[15] Tzelves L., Türk C., Skolarikos A. (2021). European Association of Urology Urolithiasis Guidelines: where Are We Going?. *Eur Urol Focus*.

[16] Xiao Y., Xiao Z. (2023). Association Between Serum Klotho and Kidney Stones in US Middle-Aged and Older Individuals with Diabetes Mellitus: Results from 2007 to 2016 National Health and Nutrition Survey. *American Journal of Nephrology*.

[17] Yuan S., Larsson S. C. (2021). Assessing Causal Associations of Obesity and Diabetes with Kidney Stones Using Mendelian Randomization Analysis. *Molecular Genetics and Metabolism*.

[18] Mao W., Zhang L., Sun S. (2022). Physical Activity Reduces the Effect of High Body Mass Index on Kidney Stones in Diabetes Participants from the 2007-2018 NHANES Cycles: a Cross-Sectional Study. *Frontiers in Public Health*.

[19] Nicolai L., Gaertner F., Massberg S. (2019). Platelets in Host Defense: Experimental and Clinical Insights. *Trends in Immunology*.

[20] Zhang Y., Meng Y., Chen M. (2023). Correlation Between the Systemic immune-inflammation Indicator (SII) and Serum Ferritin in US Adults: a cross-sectional Study Based on NHANES 2015-2018. *Annals of Medicine*.

[21] Yeap B. B., Divitini M. L., Gunton J. E. (2015). Higher Ferritin Levels, but Not Serum Iron or Transferrin Saturation, are Associated with Type 2 Diabetes Mellitus in Adult Men and Women Free of Genetic Haemochromatosis. *Clinical Endocrinology*.

[22] Kim C. H., Kim H. K., Bae S. J., Park J. Y., Lee K. U. (2011). Association of Elevated Serum Ferritin Concentration with Insulin Resistance and Impaired Glucose Metabolism in Korean Men and Women. *Metabolism*.

[23] Di X., Liu S., Xiang L., Jin X. (2023). Association Between the Systemic immune-inflammation Index and Kidney Stone: a cross-sectional Study of NHANES 2007-2018. *Frontiers in Immunology*.

[24] Capolongo G., Ferraro P. M., Unwin R. (2023). Inflammation and Kidney Stones: Cause and Effect. *Current Opinion in Urology*.

[25] Thongboonkerd V., Yasui T., Khan S. R. (2021). Editorial: Immunity and Inflammatory Response in Kidney Stone Disease. *Frontiers in Immunology*.

[26] Lontchi-Yimagou E., Sobngwi E., Matsha T. E., Kengne A. P. (2013). Diabetes Mellitus and Inflammation. *Current Diabetes Reports*.

[27] Halim M., Halim A. (2019). The Effects of Inflammation, Aging and Oxidative Stress on the Pathogenesis of Diabetes Mellitus (Type 2 Diabetes). *Diabetes & Metabolic Syndrome: Clinical Research Reviews*.

[28] Wang X., Bao W., Liu J. (2013). Inflammatory Markers and Risk of Type 2 Diabetes: a Systematic Review and meta-analysis. *Diabetes Care*.

[29] Liu N., Feng Y., Li J., Ma X., Ma F. (2022). Relationship Between the Dietary Inflammatory Index and Kidney Stone Prevalence. *World Journal of Urology*.

[30] Prasanchaimontri P., Monga M. (2020). Predictive Factors for Kidney Stone Recurrence in Type 2 Diabetes Mellitus. *Urology*.

[31] Khan S. R., Canales B. K., Dominguez-Gutierrez P. R. (2021). Randall’s Plaque and Calcium Oxalate Stone Formation: Role for Immunity and Inflammation. *Nature Reviews Nephrology*.

